# Completeness of reporting of setting and health worker cadre among trials on antenatal iron and folic acid supplementation in pregnancy: an assessment based on two Cochrane reviews

**DOI:** 10.1186/2046-4053-2-42

**Published:** 2013-06-17

**Authors:** Rachel Harper, Simon Lewin, Claire Glenton, Juan Pablo Peña-Rosas

**Affiliations:** 1 Norwegian Knowledge Centre for Health Services, Oslo, Norway; 2 Norwegian satellite of the Cochrane Effective Practice and Organisation of Care Group, Health Systems Research Unit, Medical Research Council of South Africa, Cape Town, South Africa; 3 Norwegian branch of the Nordic Cochrane Centre, Oslo, Norway; 4 Evidence and Programme Guidance, Department of Nutrition for Health and Development, World Health Organization, Geneva, Switzerland

## Abstract

**Background:**

Poor reporting of medical trials has triggered the development of trial reporting standards within the scientific community. In addition to a description of the proposed intervention, adequate information about the trial setting and the group of health workers (cadre) delivering the intervention would allow a better understanding of the generalizability of the trial findings, facilitate replication of trial interventions and assist with assessment of trials for inclusion in systematic reviews. This study aims to determine the completeness of reporting for trial setting and cadre among trials included in two Cochrane reviews on iron and folic acid supplementation for women during pregnancy.

**Methods:**

From the 81 trials included in the two Cochrane reviews, we extracted data on the trial setting, including the facility type and geographic location, facility descriptors (i.e. level of care) and population descriptors (i.e. socioeconomic status); and the cadre, including professional qualifications, training and supervision.

**Results:**

Almost all studies reported the facility type and location (96%). However, only 68% included this information in the “methods” section of the report. Facility descriptors and population descriptors were less commonly reported (26% and 54% respectively). For 34% of the trials, we found some account of the type of health worker that delivered the intervention. Only 4% of the trials reported any training procedures.

**Conclusions:**

Currently, complete reporting of setting and health worker cadre in iron and folic acid supplementation in pregnancy trials remains far from ideal, limiting assessments of the applicability of the trial findings. Trialists and journals need to ensure that this information is included in trial reports by adhering to and improving current reporting standards and by not making assumptions regarding readers’ knowledge of the context and of the intervention delivery mechanism.

## Background

Inadequate reporting of interventions in clinical trials limits the potential of health research to inform policy making and practice [1]. Assessing generalizability and critically appraising or understanding trial results, as well as exploring heterogeneous results in systematic reviews of trials, becomes a significant challenge when the intervention is described poorly. Policy makers, clinicians and program managers increasingly look to systematic reviews and evidence-based clinical guidelines to steer decisions on health interventions [2,3]. However, to implement a global guideline in a particular jurisdiction it is necessary to recognize local needs, priorities, barriers and resources. With the current status of trial reporting, authors retrieving and summarizing the evidence, as well as decision makers interpreting it, may have difficulty assessing whether an efficacious intervention could be applied in their context. Importantly, the effects of interventions may vary with health system factors, such as setting and delivering cadre [4]. More complete descriptions of the contexts or settings in which trials were implemented, and which health worker or other cadres were involved, are therefore essential [5-7]. Better descriptions of setting and health worker cadres are also necessary for reproducing the interventions in further trials and for the critical evaluation of trial results when conducting systematic reviews [8].

Guidelines such as the Consolidated Standards of Reporting Trials (CONSORT) and Transparent Reporting of Evaluations with Nonrandomized Designs (TREND) have been developed to facilitate more complete reporting of trials [9,10]. The CONSORT statement specifically recommends reporting of setting and intervention ‘with sufficient details to allow replication’. For trials intending to guide care decisions, the CONSORT extension for pragmatic trials suggests describing the population, health worker cadre and healthcare facility so as to account for variability in results due to these aspects of a trial [11]. Previous studies have investigated the extent to which these variables are reliably included in trial reports. There have been recent indications of improvement in this area, especially in the decade since the release of the CONSORT statement, but reporting is still not optimal [12].

### Objective

To determine the completeness of reporting for trial setting and cadre among trials included in two Cochrane reviews of iron and folic acid supplementation for women during pregnancy.

## Methods

We assessed all trials included in two Cochrane reviews that evaluated the effectiveness of iron and folic acid or other micronutrient supplementation for women during pregnancy. One review [13] evaluated the benefits and harms of daily intervention with iron and folic acid, while the other [14] assessed the benefits and harms of intermittent supplementation. These reviews were selected because they are typical of reviews of nutritional interventions in public health and because they were to be used in the development of a global evidence-informed guideline. We contacted the review authors for access to their trial repository, and collected the full-text versions of each published or unpublished trial report, including additional communications with the trial authors. Relevant sections of trial reports published in languages other than English were translated.

We designed a spreadsheet to evaluate each trial report based on its descriptions of setting and health worker cadre (Table 1). One author (RH) reviewed the full text of each report and of associated studies if cited, and extracted information relating to the setting - (including type of healthcare facility and its geographic location) - and additional descriptive details of the facility and the population - and information about the health worker cadre who delivered the intervention - (including level of training, any intervention-specific training, and the presence of supporting cadres or supervision). These criteria were based on sections 4 and 5 of the 2010 CONSORT statement, which gives a minimum set of recommendations for the reporting of trial participants and interventions [9]. Information was extracted from any section of the reports, noting when setting or cadre descriptions were located outside of the methods section. Incomplete descriptions, such as geographic location (for example, Toronto, Canada) without type of facility (for example, tertiary care hospital), or those that had to be inferred from sections of the text outside of the methods section, were not included in counts of satisfactory reporting. To ensure reliability, another author (SL) reviewed ten trials selected at random, and any discrepancies and irregularities in reporting were discussed with the other authors. We used simple descriptive statistics to summarize the results (Additional file 1).

**Table 1:** Trial reporting criteria investigated and numbers of trials with satisfactory reporting

**Criteria investigated**	**Descriptive points**	**Trials reporting**
Setting	Type of facility (for example, primary care clinic) and geographic location (for example, Mexico City)	68% (n = 46/68)
	Details to help clarify the setting (for example, information on care level, types of services provided, whether privately or publicly funded, characteristics of supervisory or management staff)	26% (n = 18/68)
	Description of the population receiving the intervention (for example, socioeconomic status, educational level, marital status et cetera)	54% (n = 37/68)
Health worker cadre	Level of training (professional qualifications, non-professional training or experience)	34% (n = 23/68)
	Intervention-specific training received by health workers involved in delivery of the intervention	4% (n = 3/68)
	Information regarding supporting cadres or supervision	25% (n = 17/68)

## Results

We evaluated reports for 68 of the 81 included trials. Three trials were unpublished with only abstracts or raw data available, and had no written description of the intervention. Ten reports were subsequent publications of the included trials and were grouped with the original publication for the purpose of this evaluation (Additional file 2). The papers were published between 1947 and 2009, with the majority of papers in the last 25 years. Table 1 presents a summary of the results.

### Setting and population

Ninety-six percent of the trials (n = 65/68) reported the type of healthcare facility and its geographic location. No other variable was reported as frequently, and many studies reported only these descriptive points. This information was reported in the methods section of the paper in only 68% of the papers (n = 46/65). Often this information had to be deduced from the byline, introduction, background or acknowledgments sections of the paper.

Twenty-six percent (n = 18/68) of the reports included additional detail about the facility (for example, information on care level, types of services provided, whether privately or publicly funded, characteristics of supervisory or management staff). Fifty-four percent (n = 37/68) commented on the socioeconomic characteristics of the participants.

### Cadre

Forty-three percent (n = 29/68) of the reports included some information about the health worker cadre delivering the intervention. Of these, twenty-three reports explicitly mentioned the cadre, while in the remaining six reports the delivering cadre could be reasonably deduced from other details in the report. Information about the training of primary and supporting cadres was included among 4% (n = 3/68) and 3% (n = 2/68) of the trials, respectively.

## Discussion

The reporting of setting and health worker cadre in the trials evaluated in this study was generally poor. Often, these intervention components were not stated explicitly in the methods section and had to be searched for, or deduced. This is surprising considering that numerous resources are now available for researchers designing, conducting and reporting randomized trials. For example, the CONSORT statement and checklist have been improved and extended to include abstracts, traditional Chinese medicine, sports injury treatment and pragmatic trials, among other topics. Specifically, the pragmatic trial amendment to the CONSORT Statement – the section applicable to many of the trials included in this study – discusses setting and care provider descriptions as a part of Methods and Generalizability [11]. The same is true for the TREND guidelines for the reporting of nonrandomized behavioral and public health interventions. Although many of the trials we examined were published before initiatives such as CONSORT, problems of reporting were seen across all of the trials regardless of publication date. Inadequate guidance is therefore probably not the main cause of the poor reporting seen here. Other methodological studies suggest that trial reporting is improving but also indicate that it remains far from adequate [15,16].

In a published trial report, both authors and journal editors share responsibility for ensuring the quality of reporting. There is some evidence that despite their support for reporting standards, journal editors and referees do not always adequately screen articles before publication [16,17]. Moreover, authors who try to follow the guidelines may be constrained by journal word count limitations. More recently, strategies that allow authors additional space for reporting intervention details have been made possible by electronic publication [1]. However, for the level of reporting to improve, it is crucial that both editors and authors recognize the assumptions that readers may be making; for example, a researcher in the United Kingdom may have a very different concept of a maternity ward to a policy maker in Nigeria. Currently, information necessary to inform judgments regarding the applicability of an intervention is absent from many study reports [18].

Direct contact with trial authors is another source for extended information about a trial, but this is a problematic one. Although no data were gathered from authors for this report, the researchers did attempt to contact authors of the trials included in the reviews as part of a larger study. Email addresses or phone numbers were difficult to find for all researchers and some of the primary and secondary authors were deceased or no longer in the field. Of the authors whom we were able to contact, a majority did not respond to our emails. This is therefore not an adequate, long-term solution.

Rather than adjusting current standards for reporting, such as CONSORT and TREND, perhaps we need to revisit what is understood by ‘an intervention’. In this study, the importance given to the reporting of wider health system elements related to setting and care provider appeared to be low, despite these being essential to interpreting the outcomes and assessing the generalizability of a study. This may be because trial authors regard the technical aspects of interventions - such as the dose, formula, time-scale, and delivery method of micronutrient supplements - as elements of the intervention that deserve description [13,14]. However, we would argue that wider health systems elements should be consistently considered and reported by researchers so as to inform judgments about whether and how to implement interventions within a health system.

## Conclusions

This study confirms that some elements of trial reporting in the field of public health nutrition are not complete. Authors frequently include the geographic location of a trial, but fail to supply setting and cadre details necessary for the application of study findings. Strategies to increase the opportunities within medical journal publications for authors to provide detailed intervention descriptions, for example, through the inclusion of electronic appendices or databases, may address some of the issues that promote poor reporting. Better reporting will, in turn, facilitate assessments of the applicability of trial findings and contribute to more appropriate implementation of interventions within health systems [1,19]. Improved reporting is also an ethical imperative, given that many trials are publicly funded.

## Competing interests

The authors declare that they have no competing interests.

## Authors’ contributions

RH, SL and CG designed the study. RH carried out the search for and the evaluation of articles, performed statistical analysis and drafted the manuscript. SL evaluated 10 trials as the control. JP translated trials in Spanish. SL, CG, and JP contributed to and reviewed the paper. All authors read and approved the final manuscript.

## Authors’ information

Rachel Harper, Norwegian Knowledge Centre for Health Services, Oslo, Norway. Simon Lewin, Norwegian Knowledge Centre for Health Services, Oslo, Norway; Norwegian satellite of the Cochrane Effective Practice and Organisation of Care Group; and Health Systems Research Unit, Medical Research Council of South Africa. Claire Glenton, Norwegian Knowledge Centre for Health Services, Oslo, Norway and Norwegian branch of the Nordic Cochrane Centre. Juan Pablo Peña-Rosas, Department of Nutrition for Health and Development, World Health Organization, Geneva, Switzerland. The authors alone are responsible for the views expressed in this article and they do not necessarily represent the views, decisions or policies of the institutions with which they are affiliated.

## Supplementary Material

Additional file 1 PRISMA checklist. Click here for file

Additional file 2 PRISMA flow-chart. Click here for file
