# Supplementary material for: Completeness of reporting of setting and health worker cadre among trials on antenatal iron and folic acid supplementation in pregnancy: an assessment based on two Cochrane reviews
Source: Syst Rev. 2013 Jun 17;2:42. doi: 10.1186/2046-4053-2-42 (PMC3689645; doi:10.1186/2046-4053-2-42)
Supplement: Additional file 2 — PRISMA flow-chart. [file 2046-4053-2-42-S2.doc]

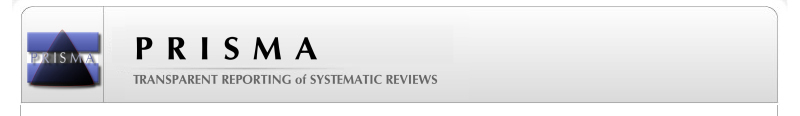
**PRISMA 2009 Flow Diagram**

**Screening**

**Included**

**Eligibility**

**Identification**

Additional records identified from the reviews
(n = 81 )

Records after duplicates removed
(n = 71 )

Records screened
(n = 71 )

Records excluded due to no published report available
(n = 3 )

Full-text articles assessed for eligibility
(n = 68 )

Full-text articles excluded
(n = 0 )

Studies included in qualitative synthesis
(n = 68 )

Studies included in quantitative synthesis (meta-analysis)
(n = N/A )
